# Supplementary material for: Efficacy and Safety of a Modified Vaccinia Ankara-NP+M1 Vaccine Combined with QIV in People Aged 65 and Older: A Randomised Controlled Clinical Trial (INVICTUS)
Source: Vaccines (Basel). 2021 Aug 3;9(8):851. doi: 10.3390/vaccines9080851 (PMC8402379; doi:10.3390/vaccines9080851)

Supplementary Table S1. Participating sites and investigators

Dr Robin Fox

Bicester Health Centre

Bicester OX26 6AT

Dr Chris Keast

The Boathouse Surgery

Pangbourne RG8 7DP

Dr Ian Binnian

Eynsham Medical Group

Eynsham OX29 4QB

Dr Simon Cartwright

The White Horse Medical Practice

Faringdon SN7 7YU Nick Thomas

Windrush Medical Practice

Witney OX28 6JS

Dr Zishan Ali

Wokingham Medical Centre

Wokingham RG40 1XS

Dr Nick Thomas

Windrush Medical Practice

Witney, OX28 6JS

Supplementary Table S2: Inclusion/exclusion criteria

Inclusion Criteria

1. Participant is willing and has capacity to provide written informed consent for participation in the study (in the Investigator's opinion)
2. Male or female adults, aged 65 years and above
3. Able and willing (in the Investigator's opinion) to comply with all study requirements
4. Willing to allow the Investigators to discuss the participant's medical history with their GP
5. Eligible to receive seasonal influenza vaccine

Exclusion Criteria

1. Any history of anaphylaxis in reaction to vaccination or history of allergic reactions likely to be exacerbated by any component of the vaccine (e.g., egg allergy)
2. Ongoing terminal illness with a life expectancy estimated to be approximately <6 months
3. Continuous use of oral anticoagulants, such as coumarins and related anticoagulants (i.e., warfarin) or novel oral anticoagulants (i.e., apixaban, rivaroxaban, dabigatran and edoxaban)
4. Any other significant disease, disorder or finding (including blood test results), which, in the opinion of the Investigators, would either put the participant at risk because of participation in the study, or may influence the result of the study
5. Participation in another clinical study of an investigational medicinal product in the 30 days preceding enrolment, or planned use during the study period
6. Prior receipt of an investigational vaccine likely to impact on interpretation of the study data
7. Receipt of annual seasonal influenza vaccine prior to enrolment (for the same influenza season participants are recruited in)
8. Not willing to comply with study procedures

Supplementary Table S3: Summary of other Baseline Variables (ITT Population)

	MVA-NP+M1 (N=432)	Placebo (N=430)	Total (N=862)
**Co-morbidities on study entry**			
Cardiovascular disease	187 (43.3%)	193 (44.9%)	380 (44.1%)
Metabolic disease	158 (36.6%)	149 (34.7%)	307 (35.6%)
Neurological disease	42 (9.7%)	22 (5.1%)	64 (7.4%)
Conditions of the eyes/ears	155 (35.9%)	162 (37.7%)	317 (36.8%)
Respiratory disease	56 (13.0%)	68 (15.8%)	124 (14.4%)
Renal/genitourinary disease	27 (6.3%)	45 (10.5%)	72 (8.4%)
Musculoskeletal disease	154 (35.6%)	146 (34.0%)	300 (34.8%)
Cancers	26 (6.0%)	39 (9.1%)	65 (7.5%)
Other	92 (21.3%)	88 (20.5%)	180 (20.9%)
**Concomitant medications**			
Antihypertensive	166 (38.4%)	175 (40.7%)	341 (39.6%)
Antidiabetic	30 (6.9%)	23 (5.3%)	53 (6.1%)
Statins	154 (35.6%)	153 (35.6%)	307 (35.6%)
Bronchodilators/Inhaled	49 (11.3%)	46 (10.7%)	95 (11.0%)
Painkillers	45 (10.4%)	37 (8.6%)	82 (9.5%)
Other[a]	165 (38.2%)	180 (41.9%)	345 (40.0%)

Supplementary Table S4

Membership of Data Sets included in Analysis

**Analysis Dataset**	**Number of Participants**		
	**MVA-NP+M1**	**Placebo**	**Total**
ITT analysis[a]	432	430	862
Safety analysis	431	429	860
Immunology sub-cohort analysis	26	24	50

Abbreviations: ITT=intent to treat

[a] Only participants in the ITT population who completed diaries were included in the efficacy analyses

Supplementary Table S5 Overall Summary of Unsolicited Adverse Events (Safety Population)

	MVA-NP+M1 (N=431)	Placebo (N=429)	Total (N=860)
Number of adverse events	108	100	208
Number (%) participants with adverse events	61 (14.2%)	59 (13.8%)	120 (14.0%)
Number of study vaccine-related adverse events[1]	46	17	63
Proportion of adverse events assessed to be study vaccine-related	42.6%	17.7%	30.9%
Number (%) participants with study vaccine-related adverse events	28 (6.5%)	11 (2.6%)	39 (4.5%)
Proportion of participants with adverse events assessed to be related	45.9%	18.6%	32.5%
Number of SAEs	17	11	28
Number (%) participants with SAEs	15 (3.5%)	11 (2.6%)	26 (3.0%)
Number of severe (Grade 3) adverse events	92	81	173
Number (%) participants with severe (Grade 3) adverse events	49 (11.4%)	45 (10.5%)	94 (10.9%)
Number (%) participants with adverse events leading to withdrawal	1 (0.2%)	1 (0.2%)	2 (0.2%)

Abbreviations: SAE=serious adverse event

1 Defined as those with a definite, probable or possible causality

Supplementary Table S6 Number of Days with Moderate or Severe Symptoms During an Influenza-like Illness Episode (Participants in the ITT Population with Available Data)

Number of Days with Moderate/Severe Symptoms	Number (%) Participants	
	**MVA-NP+M1 (N=420)**	**Placebo (N=426)**
0	351 (83.6%)	356 (83.6%)
1-6	40 (9.5%)	44 (10.3%)
7+	29 (6.9%)	26 (6.1%)
**Mean (SD) [Range]**	**1.1 (3.8) [0.0 to 44.0]**	**1.1 (3.3) [0.0 to 29.0]**
Total days of moderate/severe symptoms during an ILI	466	460
Total days follow-up	50402	50937

Supplementary Table S7: Duration of Influenza-like Illness based on Number of Episodes

	**MVA-NP+M1 (N=83)**0	**Placebo (N=94)**0	**Overall (N=177)**
Median (range) duration of symptoms per ILI episode	7.0 (1.0 to 58.0)	5.0 (1.0 to 28.0)	6.0 (1.0 to 58.0)
**Unadjusted fixed mixed effect analysis**			
	**Geometric mean**0	**P-value**	**Confidence Interval**
MVA-NP+M1 versus placebo	0.983	0.597	0.922-1.048

a N is based on number of episodes

b Estimates from the model were exponentiated, therefore the parameter estimate represents the geometric mean duration of ILI between the randomised groups[7,14-19,26,27]

Supplementary Table S8: Estimated Frequency of Influenza Infection using Historical Data on the Proportion of Influenza-like Illnesses that are caused by Influenza Virus Infection (Participants in the ITT Population with Available Data)

<table>
<tr><th colspan="6">Number (%) Participants</th></tr>
<tr><th colspan="2">MVA-NP+M1 (N=420)</th><th colspan="2">Placebo (N=426)</th><th colspan="2">Total (N=846)</th></tr>
<tr><th>Frequency of ILI</th><th>Estimated ILIs caused by Influenza</th><th>Frequency of ILI</th><th>Estimated ILIs caused by Influenza</th><th>Frequency of ILI</th><th>Estimated ILIs caused by Influenza</th></tr>
<tr><td rowspan="2">72 (17.1%)</td><td>13.0 (3.1%)a</td><td rowspan="2">79 (18.5%)</td><td>14.2 (3.3%)a</td><td rowspan="2">151 (17.8%)</td><td>27.2 (3.2%)a</td></tr>
<tr><td>8.8 (2.1%)b</td><td>9.7 (2.3%)b</td><td>18.5 (2.2%)b</td></tr>
<tr><td colspan="6">Abbreviations: ILI=influenza-like illness
a. Estimated rate of influenza-like illness attributable to influenza for all age groups 18% (5)
b. Estimated rate influenza-like illness attributable to influenza for the age 65 years and over age group 12.25%</td></tr>
</table>

Supplementary Figure S1. Proportion of Participants with Moderate or Severe Systemic Reactogenicity over the first 7 days (Safety Population); (Red- MVA-NP+M1, Blue- Placebo)

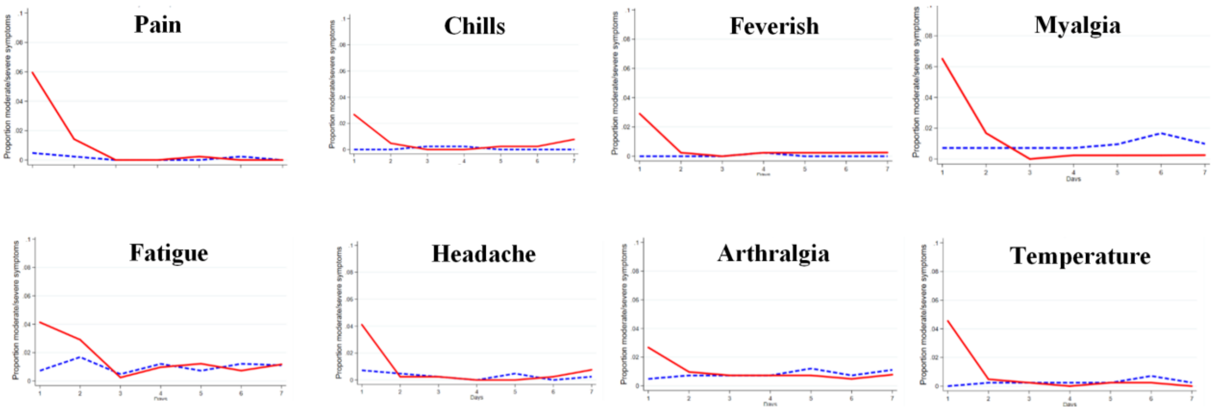

Supplementary Figure S2. ICS on a Subset of the Immunology Cohort: Day 0/21 (* p <0.01)**

INVICTUS FLU study ICS data:
IFNγ+IL-2+TNFα+ cells (% frequency of CD4+ or CD8+ T cells).

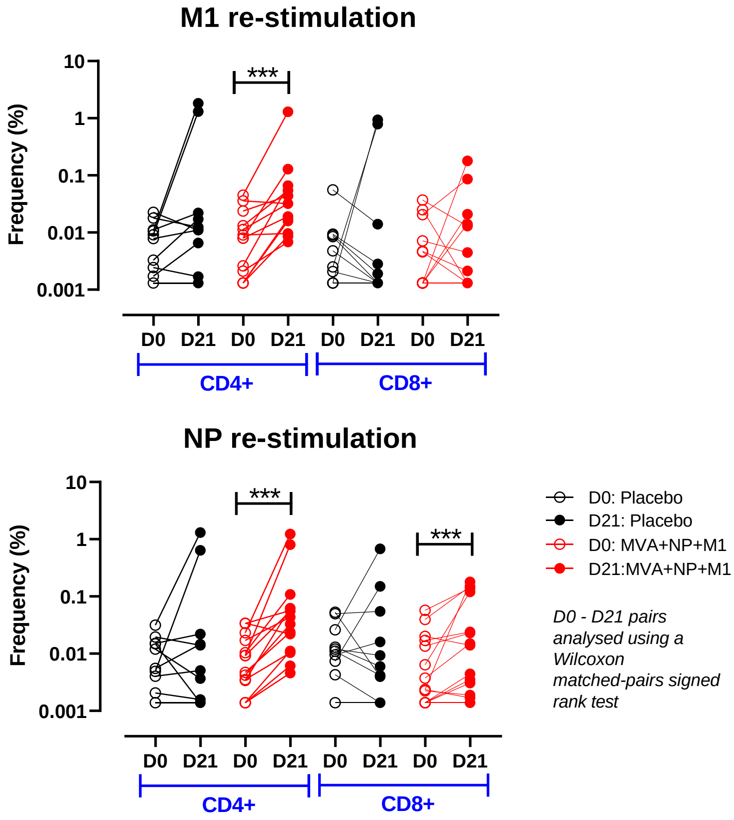

Supplement: Supplementary file 1 [file vaccines-09-00851-s001.zip › vaccines-1248857 Supplemental Material mdpi.pdf]
